# Multifunctional Composite Microcapsules for Oral Delivery of Insulin

**DOI:** 10.3390/ijms18010054

**Published:** 2016-12-28

**Authors:** Shaoping Sun, Na Liang, Xianfeng Gong, Weiwei An, Yoshiaki Kawashima, Fude Cui, Pengfei Yan

**Affiliations:** 1 Key Laboratory of Chemical Engineering Process and Technology for High-Efficiency Conversion, School of Chemistry and Material Science, College of Heilongjiang Province (Heilongjiang University), Heilongjiang University, Harbin 150080, China; sunshaoping111@163.com (S.S.); gongxianfeng@sina.com (X.G.); 2 College of Chemistry and Chemical Engineering, Harbin Normal University, Harbin 150025, China; 3 Institute of Tumor Research, Harbin Medical University, Harbin 150080, China; 13766855349@139.com; 4 School of Pharmaceutical Science, Aichi Gakuin University, Nagoya 470-0195, Japan; sykawa123@163.com; 5 School of Pharmacy, Shenyang Pharmaceutical University, Shenyang 110016, China; syphucuifude@163.com

**Keywords:** insulin, sodium deoxycholate, HP55, PLGA nanoparticles, microcapsules

## Abstract

In this study, we designed and developed a new drug delivery system of multifunctional composite microcapsules for oral administration of insulin. Firstly, in order to enhance the encapsulation efficiency, insulin was complexed with functional sodium deoxycholate to form insulin-sodium deoxycholate complex using hydrophobic ion pairing method. Then the complex was encapsulated into poly(lactide-*co*-glycolide) (PLGA) nanoparticles by emulsion solvent diffusion method. The PLGA nanoparticles have a mean size of 168 nm and a zeta potential of −29.2 mV. The encapsulation efficiency was increased to 94.2% for the complex. In order to deliver insulin to specific gastrointestinal regions and reduce the burst release of insulin from PLGA nanoparticles, hence enhancing the bioavailability of insulin, enteric targeting multifunctional composite microcapsules were further prepared by encapsulating PLGA nanoparticles into pH-sensitive hydroxypropyl methyl cellulose phthalate (HP55) using organic spray-drying method. A pH-dependent insulin release profile was observed for this drug delivery system in vitro. All these strategies help to enhance the encapsulation efficiency, control the drug release, and protect insulin from degradation. In diabetic fasted rats, administration of the composite microcapsules produced a great enhancement in the relative bioavailability, which illustrated that this formulation was an effective candidate for oral insulin delivery.

## 1. Introduction

Multiple subcutaneous injection of insulin remains the common approach for the treatment of insulin-dependent diabetic patients [1,2]. Nevertheless, there are many major adverse effects with this therapy, including hypoglycemia, allergy, insulin resistance, and edema [3]. In addition, multiple and frequent injection of insulin is painful and inconvenient, which leads to poor patient compliance. It was reported that insulin administered via subcutaneous route is delivered to the peripheral circulation, so it cannot mimic the physiological hypoglycemic mechanism by directly delivering insulin into liver after absorption in normal subjects [4,5]. All of these events have provoked numerous attempts to develop a safe and effective noninvasive route for insulin delivery.

In recent decades, oral delivery of insulin has been widely investigated because it can minimize the risk of hypoglycemia and obtain high patient compliance. In addition, it was reported that oral delivery is considered as a favorable and alternative route for insulin because it undergoes a hepatic bypass before entering the circulation, so it can simulate the effect of pancreas-secreted insulin in terms of inhibiting hepatic gluconeogenesis and hepatic glucose output [6,7,8,9].

Many different strategies have been attempted to develop a biologically active oral insulin formulation, such as the reverse micelle-solvent evaporation method, emulsion solvent diffusion method, and nanoprecipitation-solvent displacement method. In those methods, insulin molecules are usually exposed to solvent, heat, and high surface tension which results in the degradation of insulin. Some of these methods are also limited because of their low encapsulation efficiency and unacceptable burst release of hydrophilic peptidic drugs [10]. In order to overcome these aforementioned obstacles and to enhance the oral bioavailability of insulin, biodegradable polymeric nanoparticles (NPs), such as poly(lacticacid), poly(glycolic acid), and their copolymers poly(lactide-*co*-glycolide) (PLGA) NPs, have been widely studied by our group [11,12]. Additionally, in recent years, there have been several techniques developed to provide the preparation of stable and efficient proteins encapsulation including the microbubbling process, coaxial electrohydrodynamic spraying method, and electrohydrodynamic atomization technology [10,13,14].

However, the delivery of insulin via the oral route is a great challenge because of acidic and enzymatic instability and the poor absorption of insulin. The stomach forms the boundary between the small intestine and the external environment, which is the first obstacle for oral delivery of insulin. In order to prevent insulin from contacting acidic medium in the stomach, special pH sensitive NPs were prepared to provide the required protection [15,16,17,18,19,20]. Among the enteric polymers, hydroxypropyl methyl cellulose phthalate (HP55, pKa = 5.5) was used as a promising material to prepare enteric targeting formulations for its special characteristics. HP55 is stable in acidic gastric fluid but degradable in enteric conditions. Interestingly, HP55 can be dissolved in polar organic solvents such as methanol, ethanol, acetone, or their mixture with water, therefore, HP55 microcapsules including protein or peptide can be prepared at low temperature by organic spray-drying method [21]. In this study, the organic spray-drying method could prevent the degradation of insulin.

The epithelial cells of the gastrointestinal tract are tightly bound together to form tight junctions, which inhibit the passage of insulin and its subsequent absorption [7]; this is the secondary obstacle for oral delivery of insulin. In order to enhance absorption of insulin, absorption enhancers such as trisodium citrates, bile salts, ethylene diamine tetraacetic acid, and chitosan were widely studied to open the tight junctions of the intestinal epithelium [22,23,24,25]. It was reported that sodium deoxycholate can promote the absorption by paracellular and transcellular transport pathways [26,27]. In addition, sodium deoxycholate is produced in the liver, after being released into the duodenum, it can be reabsorbed from the intestinal lumen and returned to the liver via the portal vascular system [28]. The special selective reabsorption character can potentially be used to enhance the absorption of insulin. Moreover, sodium deoxycholate complexed with insulin can increase the liposolubility, and therefore increase the encapsulation efficiency, of insulin in polymeric NPs [11].

In this study, we developed a multifunctional formulation that is able to deliver the entrapped insulin to the small intestine and promote the absorption of insulin. Insulin was first complexed by ionic pairing method with functional sodium deoxycholate, and then encapsulated into PLGA NPs with high encapsulation efficiency with the help of its increased liposolubility. To further avoid the rapid acidic and enzymatic degradation and increase oral relative bioavailability, insulin loaded NPs were encapsulated into a pH sensitive enteric polymer HP55 to form an enteric targeting multifunctional composite microcapsules by organic spraying method. The physicochemical characterizations, in vitro release of insulin, and behavior of the prepared particles and their efficiency in lowering the blood glucose in diabetic rats were studied.

## 2. Results and Discussion

### 2.1. Preparation and Characterization of the Particles

Sodium deoxycholate is an amphiphilic biological compound with a hydrophobic α-side and a hydrophilic β-side. It is synthesized in the liver, from where it is carried via the bile duct to the small intestine, and returned to the liver by absorption through the bile acid transporter [29]. The enterohepatic circulation of the sodium deoxycholate suggests that the introduction of sodium deoxycholate into Ins-SD-Comp can result in the cycling of insulin in the enterohepatic circulation, which could provide sustained release into the bloodstream.

**Figure 1:**
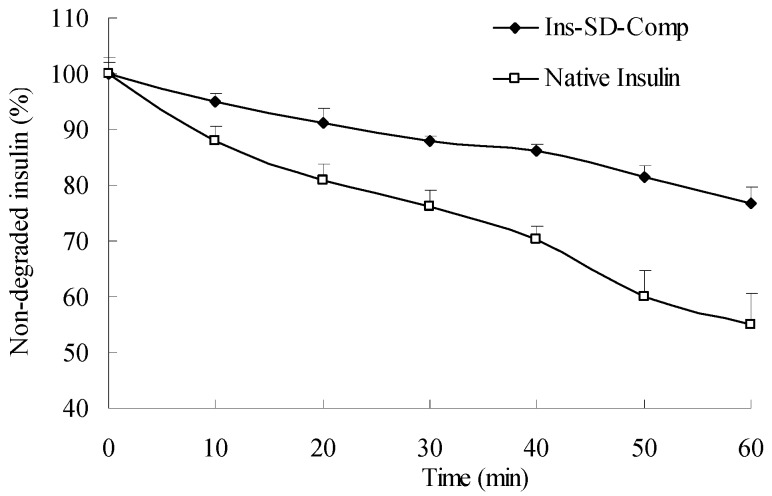
Enzymatic degradation profiles of insulin as a function of time in α-chymotrypsin.

From Figure 1, it was observed that insulin was degraded rapidly. Only about 55% of insulin remained on incubation with α-chymotrypsin within 60 min for native insulin, and about 76.8% of insulin remained for the complex. This result can be explained as follows: due to the natural physicochemical properties of sodium deoxycholate, the insulin and sodium deoxycholate molecules formed a more lipophilic complex by hydrophobic ion pairing, which may reduce the accessibility of proteases to insulin. In addition, due to the increased portioning coefficient (increased by two orders of magnitude compared with free insulin), Ins-SD-Comp can be encapsulated into PLGA NPs with high encapsulation efficiency. The encapsulation efficiency of Ins-SD-Comp was about two times greater than that of free insulin (94.2% vs. 43.6%). The insulin loading content was calculated as 4.54%.

**Figure 2:**
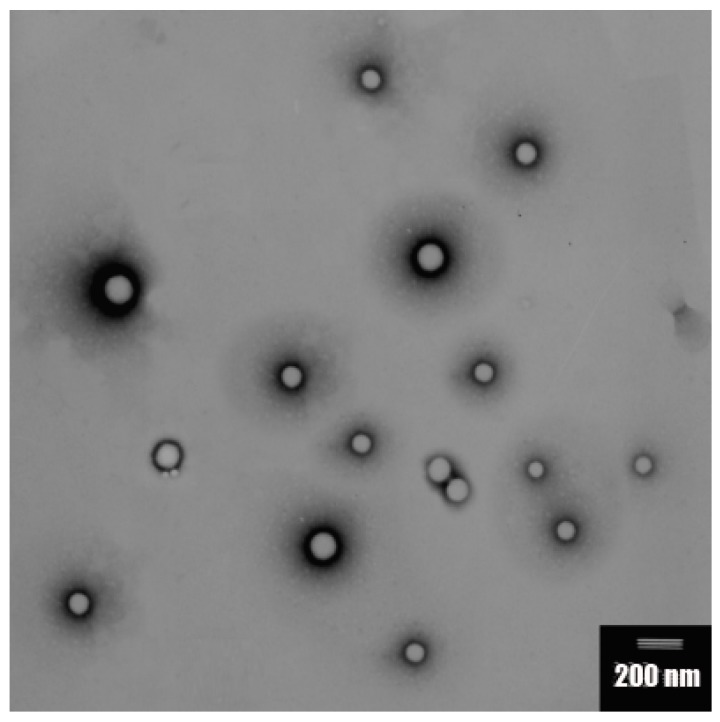
Transmission electron microscopy of Ins-SD-Comp loaded PLGA NPs.

**Figure 3:**
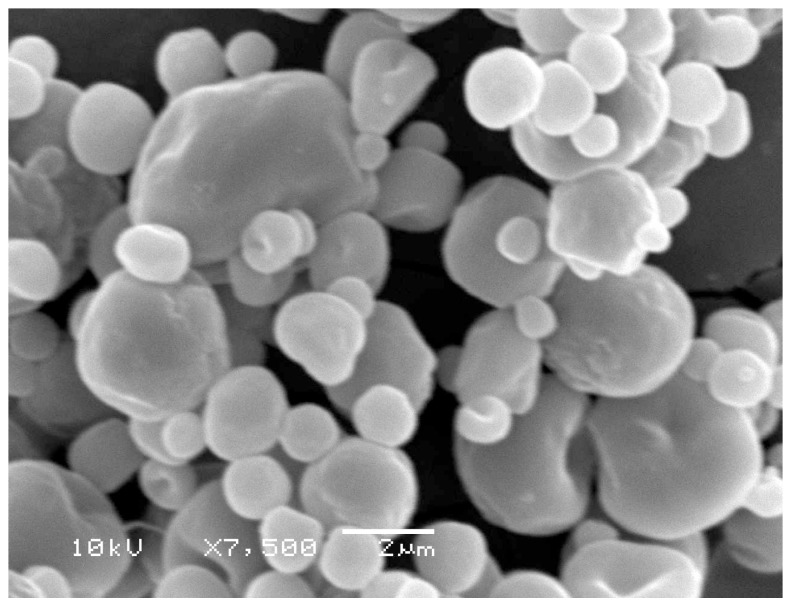
Scanning electron microscopy of multifunctional composite microcapsules.

The zeta potential of the Ins-SD-Comp PLGA NPs was −29.2 mV. The mean particle size of Ins-SD-Comp PLGA NPs was about 168 ± 11 nm. The polydispersity index of 0.072 ± 0.021 (*n* = 3) indicated a narrow size distribution. Morphology of the PLGA NPs was studied by TEM method. From Figure 2, it can be observed that the particles were monodisperse spherical. In this study, Ins-SD-Comp loaded PLGA NPs were further encapsulated into HP55 to develop an enteric targeting formulation. It was observed from Figure 3 that the composite microcapsules displayed a nearly spherical shape and smooth surface, with diameters ranging from 1 to 5 µm. Furthermore, numerous NPs could be detected within the microcapsules′ void cores following incubation in the pH 6.8 release medium over 4 h. The final production yield was about 87.8%, with drug recovery of 82.6%. In this study, a nitrogen circulating spray-dryer was applied to prepare the composite microcapsules. The suspension can be spray-dried at low temperature due to the fact that organic solvent can be used in this system. In addition, the organic solvent can be evaporated immediately, for the droplet was very small when suspension was sprayed out from the nozzle. The prepared composite microcapsules were collected soon in the cyclone separator, and insulin cannot be degraded in such a short time. This hypothesis was confirmed in Section 2.4.

### 2.2. In Vitro Release Kinetic Experiment

**Figure 4:**
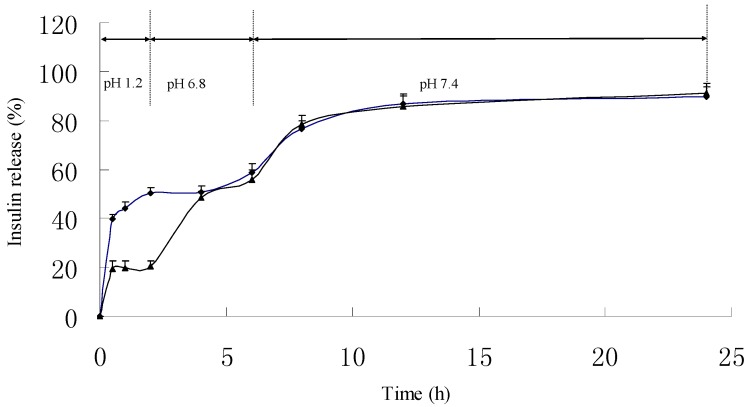
In vitro release profiles of insulin from the multifunctional composite microcapsules (▲) and PLGA NPs (◆) in gradual pH-changing buffers (*n* = 3).

**Figure 5:**
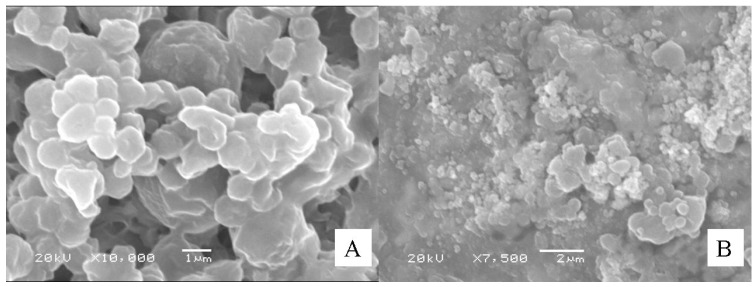
Scanning electron microscopy of the centrifuged multifunctional composite microcapsules at determined points during in vitro release ((**A**) 2 h, pH 1.2; (**B**) 6 h, pH 6.8).

As shown in Figure 4, it can be observed that the PLGA NPs displayed an unfavorable pH-sensitive release profile in dissolution medium. About 50.2% of insulin was released in pH 1.2 dissolution medium within the first 2 h. When the PLGA NPs were encapsulated into HP55 to prepare composite microcapsules as a multifunctional delivery system, the initial release of insulin dramatically reduced to 20.3% in pH 1.2 dissolution medium within the first 2 h. During preparation of the composite microcapsules, insulin may adhere on the surface, which can explain about 20.3% burst release of insulin. As expected, we also found that the insulin release profiles of the PLGA NPs and multifunctional composite microcapsules were similar at pH 6.8 from the forth hour, the cumulative insulin release from PLGA NPs and the composite microcapsules after 6 h were about 58.7% ± 3.53% and 55.8% ± 1.91%, respectively. Indeed, the tested multifunctional composite microcapsules remained intact in acidic environment during the experimental process. When pH was changed to 6.8, the outer layer of the composite microcapsules was dissolved, and then the NPs were released. The HP55 was functional as the carrier to protect the insulin released in an acidic stomach environment, while the sodium deoxycholate has the ability to open tight junction and enhance the permeation of NPs and the released insulin. Figure 5A showed that the multifunctional composite microcapsules remained intact after being incubated in pH 1.2 dissolution medium for 2 h. After being incubated in pH 6.8 dissolution medium for 4 h, the enteric HP55 was dissolved, and the PLGA NPs were released from the multifunctional composite microcapsules rapidly as shown in Figure 5B. It can be speculated that the release of insulin was controlled by both the outer layer of multifunctional composite microcapsules and the PLGA NPs.

### 2.3. Studies of Disposition of the Particles in the Gastrointestinal Tract and Absorption Mechanism in Ileum

**Figure 6:**
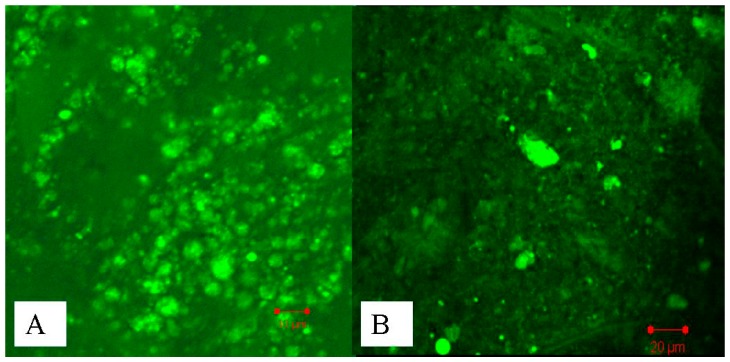
Disposition of C-6-loaded composite microcapsules in the gastrointestinal tract at different time after oral administration to normal rats ((**A**) 2 h, duodenum; (**B**) 4 h, ileum).

**Figure 7:**
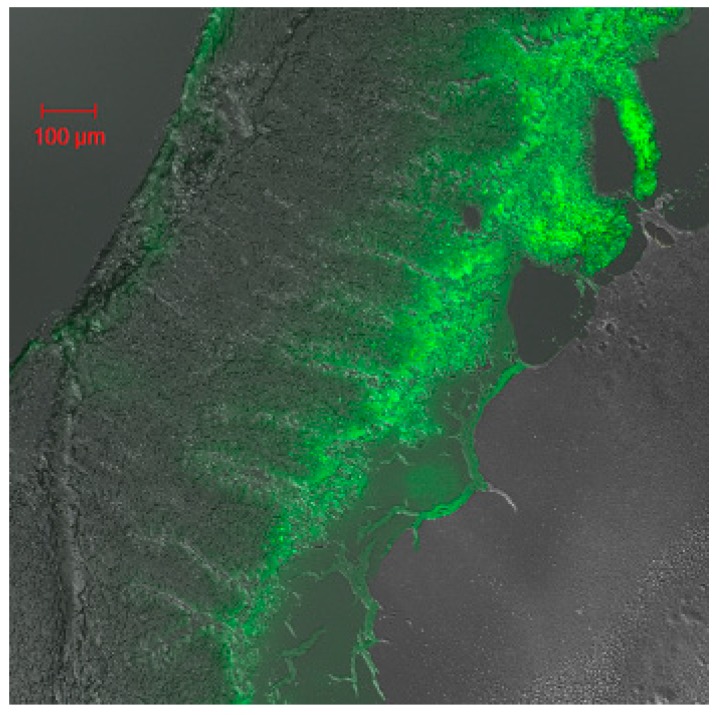
Confocal laser scanning microscopy (CLSM) image of Peyer′s patches cross-sections prepared 4 h after oral administration of C-6-loaded composite microcapsules.

The results of in vitro release illustrated that the multifunctional composite microcapsules could remain intact in the stomach (0–2 h). However, when the multifunctional composite microcapsules reached the small intestine from the second hour, most of PLGA NPs were released in the next 4 h, and insulin was then released from the PLGA NPs. In order to confirm our hypothesis, CLSM studies were performed. Figure 6A showed that most of composite microcapsules remained intact in the duodenum 2 h after oral administration, and most of the PLGA NPs were released from the composite microcapsules in the ileum 4 h after oral administration (Figure 6B). This indicated that the release of insulin from multifunctional composite microcapsules could be controlled both by microcapsules and PLGA NPs. It has been reported that PLGA NPs can be directly taken up by Peyer′s patches in the ileum [30], which was confirmed by Figure 7. In addition, after insulin was released and reached the colon, it can also be absorbed because of the low activity of proteolytic enzymes in the colon. Thus, this pattern of release profile is ideal for oral administration of insulin.

### 2.4. Biological Activity of Insulin

**Figure 8:**
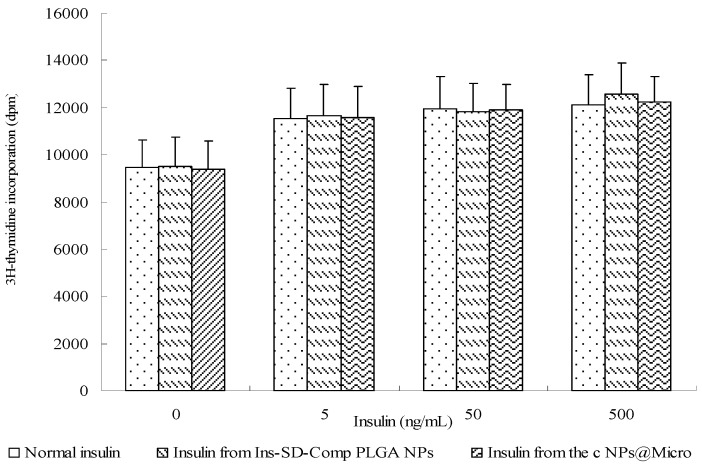
Biological activity of normal insulin, insulin extracted from the Ins-SD-Comp PLGA NPs, and insulin extracted from multifunctional composite microcapsules. Data are the mean ± S.D. (*n* = 6).

During the preparation of multifunctional composite microcapsules, several chemicals and solvents including acetone, ethanol, and dilute HCl were used. It is important to determine whether insulin maintains its biological activity or not, because insulin is readily destroyed during the preparation process. Insulin acts as a growth factor, meanwhile, it can control glucose levels. NIH 3T3 mouse fibroblasts are insensitive to insulin but are responsive to IGF-1 [31]. Insulin and IGF regulate the cellular functions by both overlapping receptor and postreceptor signaling pathways [32]. Therefore, we used these cells to test the biological activity of insulin extracted from the samples compared with that of normal insulin. In control cells, (3H)thymidine uptake was 9485.7 ± 1125.9 dpm (Figure 8). The potency of cells treated with extracted insulin was similar to that of normal insulin, which showed insulin retained its bioactivity during the preparation process. It is probably due to the short contact time and the protection of PLGA NPs.

### 2.5. Hypoglycemic Effect of the Multifunctional Composite Microcapsules

**Figure 9:**
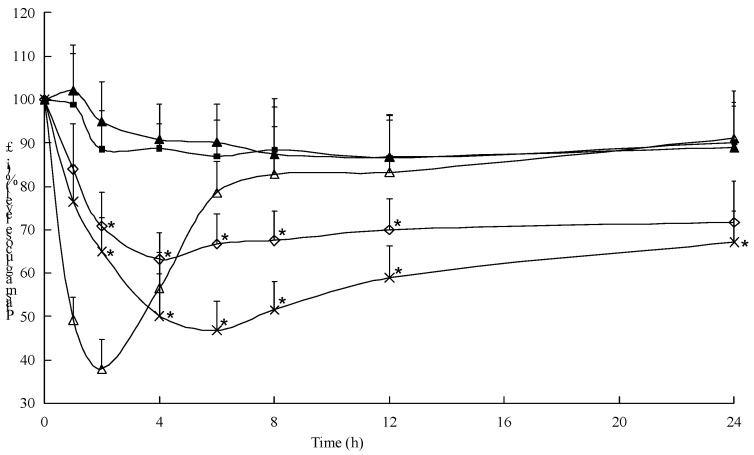
Blood glucose level with time after administration of various samples to diabetic rats. (∆) subcutaneous injection of insulin solution (1.5 IU/kg), (×) oral administration of multifunctional composite microcapsules (20 IU/kg), (◇) PLGA NPs (20 IU/kg), (■) native insulin (20 IU/kg), and (▲) saline. Data represent the mean ± S.D. (*n* = 6). * *p* < 0.05, compared with the group of oral administration of native insulin.

From Figure 9, it was observed that the oral insulin solution (20 IU/kg) and distilled water did not result in significant reduction of glycemia. The initial increase of blood glucose levels in both of the groups was related to the stress of administration. Oral administration of Ins–SD-Comp PLGA NPs resulted in considerable reduction of the blood glycemic level as compared to oral insulin solution, although there was a big burst release of insulin. Two hours after oral administration of Ins-SD-Comp PLGA NPs, the blood glucose level decreased to 70.8% ± 7.68%, and then the level decreased to 63.2% ± 6.92% at 4 h and maintained a sustained reduction. Initial burst release of Ins-SD-Comp from PLGA NPs in the intestinal lumen was responsible for the first physiological effect. Then, after arrival to the ileum and colon, polymer NPs and released insulin could be taken up and absorbed, resulting in a prolonged hypoglycemic effect compared with oral administration of insulin solution. A *t*-test with the significance level of *p* < 0.05 showed that there was a significant difference between the preparations and the control groups.

Interestingly, oral administration of multifunctional composite microcapsules showed a significant hypoglycemic effect in diabetic rats. The observed hypoglycemic efficacy of the composite microcapsules can be illuminated by the synergistic effects of this multifunctional delivery system. The enteric composite microcapsules served as a platform for encapsulation of Ins-SD-Comp and rendered insulin more stable against the harsh environment of the stomach. The absorption enhancing characteristics of sodium deoxycholate may induce the opening of epithelial tight junctions between enterocytes, and allow the transport of insulin. Furthermore, insulin could be potentially reabsorbed from the intestinal lumen and returned to the liver via the portal vascular system because of the enterohepatic circulation property of sodium deoxycholate. It was reported that HP55 can markedly improve insulin resistance against acid degradation [33]. As such, the multifunctional composite microcapsules can well control the insulin release in desired region of the gastrointestinal tract. After arriving at the small intestine, most of the PLGA NPs were released and reached a high particle gradient concentration, so the intact NPs could be taken up by Peyer′s patches in the small intestine [30]. By virtue of the permeation enhancing property of sodium deoxycholate, the released insulin could also be absorbed via the paracellular pathway, as well as by transcytosis of the encapsulated protein through the intestinal enterocytes and Peyer′s patches [34,35]. These effects may act synergistically to enhance and prolong the efficacy of insulin delivered by the multifunctional delivery system.

The multifunctional composite microcapsules thus helped in improving the in vivo response for 24 h from one single application, and the relative bioavailability was about 16.1% for the multifunctional composite microcapsules while for free insulin it was less than 0.5%.

## 3. Materials and Methods

### 3.1. Materials and Animals

Porcine insulin (28.7 IU/mg) and streptozocin (STZ) were purchased from Sigma-Aldrich (St. Louis, MO, USA). Sodium deoxycholate and poly(lactic-*co*-glycolic acid) (PLGA 75/25, molecular weight of 20 kDa) were purchased from Wako Pure Chemical Industries, Ltd. (Osaka, Japan). Hydroxypropyl methyl cellulose phthalate (HP55, with designed solubility above pH 5.5) was obtained from Shin-Etsu Chemical Co., Ltd. (Tokyo, Japan). Polyvinyl alcohol (PVA-403) was kindly supplied by Kuraray Co., Ltd. (Osaka, Japan). All other reagents were of analytical grade.

Male Wistar rats weighing 200 ± 20 g, 12–13 weeks old, were provided by Aichi Gakuin University. The study protocol was reviewed and approved by the Institutional Animal Care and Use Committee (11 March 2011, No. 1103113203), Aichi Gakuin University, Japan.

### 3.2. Preparation of Insulin-Sodium Deoxycholate Complex (Ins-SD-Comp) and the PLGA NPs

Ins-SD-Comp and Ins-SD-Comp PLGA NPs were prepared according to the method described previously [24,25]. Ins-SD-Comp and Ins-SD-Comp PLGA NPs were produced by hydrophobic ion pairing method and emulsion solvent diffusion method, respectively. Briefly, 20 mL of sodium deoxycholate (1 mg/mL) solution was added dropwise into 45 mg (pH 4.0, 40 mL) of insulin solution under magnetic stirring. The complex was collected by centrifugation at 14,000 rpm for 15 min, washed with distilled water for three times, and then lyophilized into powder. Subsequently, Ins-SD-Comp was loaded into PLGA NPs. More specifically, 5 mg of insulin-equivalent Ins-SD-Comp and 100 mg of PLGA were dissolved in acetone with a small quantity of 0.01 mol/L HCl as oil phase. Then the oil phase was dropped into 100 mL of aqueous solution containing 1% of PVA with constant stirring (400 rpm). The PLGA NPs were recovered by ultracentrifugation for 10 min (20,000 rmp, 4 °C) and washed with distilled water for three times, followed by lyophilization. The entrapped insulin fraction was calculated based on the initial amount of insulin added and the concentration of insulin in the supernatant. The encapsulation efficiency (*EE*%) of insulin and insulin loading content (*IL*%) were calculated from the following equations:

(1) $$EF\%=(W_{\mathrm{init}}-W_{\sup})/W_{\mathrm{init}}\times 100\%$$

(2) $$IL\%=(W_{\mathrm{init}}-W_{\sup})/W_{\mathrm{np}}\times 100\%$$

where *W*_init_, *W*_sup_, and *W*_np_ represent the initial amount of insulin added, amount of insulin in the supernatant, and weight of nanoparticles, respectively.

Insulin content was quantified using HPLC method. The HPLC system was equipped with a Hitachi (Hitachi, Ltd., Tokyo, Japan) pump L-7110, a Hitachi UV-Vis Detector L-7420, an ODS C 18 column (5 µm, 150 mm × 4.6 mm) and a thermostated column compartment. The HPLC mobile phase for insulin determination in this paper consisted of a premixed isocratic mixture of 0.2 M sodium sulfate anhydrous solution adjusted to pH 3.0 with phosphoric acid and acetonitrile (70:30, *v*/*v*). The injection volume of the test samples was 20 µL. Samples were eluted at a flow rate of 1.0 mL/min. The column temperature was kept at 35 °C and the detection wavelength was 214 nm. Linear calibration curve (Area = 18,092 × concentration − 27,350) was obtained in the concentration range of 5 and 100 µg/mL with a correlation coefficient (*r*) of 0.9999. All the insulin concentrations were calculated by interpolation from the standard curve.

### 3.3. Enzymatic Degradation Studies

The stability of the complex against enzymatic degradation was carried out as follows. Insulin (100 µL, 16.2 µmol/L) and an equivalent amount of Ins-SD-Comp solution were prepared by dispersing them in 2-(4-(2-Hydroxyethyl)-1-piperazinyl)ethanesulfonic acid buffer (50 mmol/L, pH 7.4) respectively. Then α-chymotrypsin (10 µL, 1.5 µg) was added and the solution was incubated at 37 °C. Aliquots of the solutions were withdrawn and the enzyme activity was terminated by adding 0.1% trifluoroacetic acid at the present time points. The samples were then analyzed using HPLC to determine the amount of insulin.

### 3.4. Preparation of Multifunctional Composite Microcapsules

**Figure 10:**
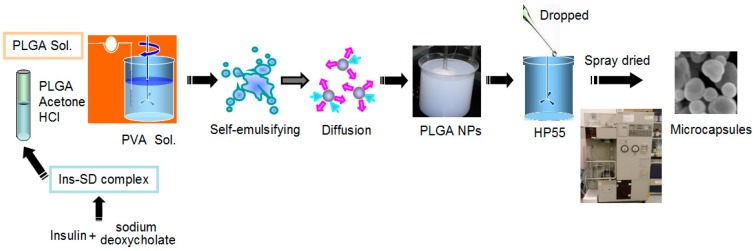
Diagram of the preparation of multifunctional composite microcapsules. Ins-SD complex, insulin-sodium deoxycholate complex; PVA, polyvinyl alcohol; PLGA, poly(lactide-*co*-glycolide); NPs, nanoparticles, HP55, hydroxypropyl methyl cellulose phthalate.

One hundred milligram of the lyophilized NPs were suspended into 1 mL of water, and the suspension was introduced into 3 mL (50 mg/mL) of HP55 methanol solution. The suspension was mixed using a stirring plate and spray-dried at inlet temperature of 55 °C, outlet temperature of 37 °C, with spray pressure of 0.1 MPa and feeding rate of 10 mL/min in a nitrogen circulating spray-dryer (GS-310 Yamato Labotech., Tokyo, Japan). The powder was collected in the cyclone separator and stored in a 4 °C refrigerator. The process of preparing multifunctional composite microcapsules was shown in Figure 10. The production yield (*PY*%) and drug recovery (*DR*%) were calculated using following equations:

(3) $$PY\%=W_{P}/W_{\mathrm{HP}55-\mathrm{nano}}\times 100\%$$

(4) $$DR\%=W_{\mathrm{ins}-\mathrm{micro}}/W_{\mathrm{ins}-\mathrm{nano}}\times 100\%$$

where *W*_p_, *W*_HP55__-nano_, *W*_ins-micro_, and *W*_ins-nano_ represent the amount of final product, total amount of HP55, and nanoparticles added initially, the amount of insulin extracted from multifunctional composite microcapsules, and the amount of insulin extracted from PLGA nanoparticles, respectively.

### 3.5. Characterization of the Particles

The particle size, polydispersity index (PDI), and zeta potential of PLGA NPs were assayed by photon correlation spectroscopy using a Zetasizer Nano-ZS90 (Malvern Instruments, Worcestershire, UK) at 25 °C. Particle size was represented by intensity distribution. The lyophilized PLGA NPs samples were suspended in distilled water before measurement. The morphologies of particles were determined by transmission electron microscopy (TEM, Hitachi JEM-100 CXII) or scanning electron microscopy (SEM, JSM 5600LV, Jeol, Tokyo, Japan). For TEM, an aqueous droplet of NPs suspension was immobilized on copper grids and negatively stained with phosphotungstate solution (2%, *w*/*v*), then dried at room temperature. For SEM, the multifunctional composite microcapsules were fixed on metallic studs with double-sided conductive tape and coated with platinum by a sputter coater (JFC-1300, Jeol) for 40 s in a vacuum environment at the current intensity of 40 mA.

### 3.6. In Vitro Release Kinetic Experiment

The particles were evaluated for in vitro release kinetics of insulin in simulated dissolution media in triplicate. The gradual pH changing buffer system was selected according to the normal variations of pH and general transit time along the gastrointestinal tract from the stomach (pH 1.2, 0–2 h) and proximal small intestine (pH 6.8, 2–6 h) to the colon region (pH 7.4, 6–12 h), as previously reported [36]. Briefly, test tubes containing samples were incubated in a thermostatic-oscillating water bath at 37 °C and shaken at 100 rpm. At a preset time point, 100 µL of suspension was withdrawn and centrifuged for 10 min (20,000 rpm, 4 °C), the corresponding concentration in the supernatant was determined by the HPLC method, and the total amount of insulin released from the particles was calculated accordingly. Some of the centrifuged samples at determined time point were dried and observed with SEM to study release behavior of NPs from the multifunctional composite microcapsules.

### 3.7. Biological Activity of Insulin

NIH 3T3 cells were seeded into a 24-well tissue culture plate (BD, Franklin Lakes, NJ, USA) at the density of 1 × 10^3^/mL. The Dulbecco′s modified Eagle′s medium supplemented with 10% fetal bovine serum and 1% penicillin–streptomycin solution was utilized as the cell culture medium. A determined amount of free insulin or insulin extracted from the samples were added to the cells, and each culture was cultivated at 37 °C for 24 h in a humidified atmosphere containing 5% CO_2_. Cell growth was assessed by the (3H)thymidine ((methyl-1′-2′-3′Hthymidine (1.40 TBq/mmol)); GE Healthcare UK Ltd., Little Chalfont, UK) incorporation assay. Radioactivity was counted with a scintillation counter (MicroBeta TriLux; PerkinElmer Inc., Waltham, MA, USA).

### 3.8. Studies of Disposition of the Particles and Absorption Mechanism in the Gastrointestinal Tract

Coumarin-6 (C-6) loaded composite microcapsules were prepared using the same preparation procedures as aforementioned with a weight ratio of about 2% (C-6:PLGA:HP 55 = 1:20:30).

Normal rats were fasted overnight and gavaged with C-6-loaded composite microcapsules. The rats were sacrificed at 2, 4, and 12 h after the oral administration. The duodenum, ileum, and colon were isolated and opened longitudinally. To observe the C-6-loaded composite microcapsules’ fate in the gastrointestinal tract, the duodenum, ileum, and colon were imaged by confocal laser scanning microscopy (CLSM) on an CLSM 510 system (Carl Zeiss Meditec AG, Jena, Germany) equipped with a laser operating at 488 nm for fluorescence excitation. To study the absorption mechanism of the multifunctional composite microcapsules, the freshly excised ileum were cryofixed in Tissue-Tek^®^ compound, sectioned (10 µm in thickness) using a cryomicrotome (Leica CM; Wetzlar, Germany), and also imaged by the CLSM.

### 3.9. In Vivo Evaluation on Diabetic Rats

The animals were kept under 12 h light/12 h dark cycles and were fed with a standard laboratory rodent diet in pellets form. The animals were fasted overnight and induced diabetics by single intraperitoneal injection of 65 mg/kg streptozotocin (STZ) in citrate buffer at pH 4.5, as previously described [37]. Seven days after streptozotocin treatment, rats with symptoms of frequent urination, loss of weight, and the fasted blood glucose levels higher than 16.67 mmol/dL were selected as diabetic rats. During the experiments, blood glucose levels were determined by monitoring the blood taken from the retroorbital plexus using the Mutarotase-GOD method (Glucose C2, Wako Pure Chemical Industries, Ltd.).

According to this method, 3 mL of glucose reagent was mixed with 20 µL of plasma, and incubated at 37 °C for 5 min. The absorbance values of plasma samples were measured against the reagent blank at 505 nm using Hitachi U-3900 UV-visible Spectrophotometer (Hitachi, Ltd., Tokyo, Japan).

For the hypoglycemic effect experiment, all the diabetic rats were fasted overnight with free access to water before testing. Thirty rats were randomly separated into five groups with six rats per group. The following formulations were intragastrically administered to rats by a single oral gavage: (1) distilled water (control group); (2) insulin solution (20 IU/kg); (3) PLGA NPs (20 IU/kg) in distilled water; and (4) the multifunctional composite microcapsules (20 IU/kg) in distilled water. Insulin solution (1.5 IU/kg) was administered subcutaneously to take its bioavailability as 100%. Blood samples were obtained from retroorbital plexus at different time points and centrifuged at 4000 rpm for 5 min to get the plasma.

The pharmacological relative bioavailability (RB) of the formulations to the subcutaneous insulin solution was calculated by the ratio of the respective areas above the plasma glucose level-time curves (AACs) corrected by the administered oral and subcutaneous doses as follows. The area was calculated by the trapezoidal rule.

(5) $$RB\%=AAC_{\mathrm{Oral}}/AAC_{\mathrm{SC}}\times Dose_{\mathrm{SC}}/Dose_{\mathrm{Oral}}\times 100\%$$

### 3.10. Statistical Analysis

Each experiment was performed in triplicate. Values were expressed as mean ± standard deviation (SD). Statistical data analysis was performed using the Student′s *t*-test with *p* < 0.05 as the level of significance.

## 4. Conclusions

The present study includes a successful preparation and characterization of the multifunctional composite microcapsules in vitro and in vivo. The drug delivery system showed high insulin encapsulation capacity with ideal pH-sensitive sustained release of insulin. Furthermore, in vivo results showed a pronounced hypoglycemic effect with improved insulin bioavailability in diabetic rats, which indicated significant intestinal absorption of insulin. Thus, the multifunctional composite microcapsules formulation might be employed as a promising carrier for oral administration of insulin.
